# Let's (Tik) Talk About Fitness Trends

**DOI:** 10.3389/fpubh.2022.899949

**Published:** 2022-07-11

**Authors:** Valdemar Štajer, Ivana M. Milovanović, Nikola Todorović, Marijana Ranisavljev, Saša Pišot, Patrik Drid

**Affiliations:** ^1^Faculty of Sport and Physical Education, University of Novi Sad, Novi Sad, Serbia; ^2^Institute for Kinesiology Research, Science and Research Centre Koper, Koper, Slovenia

**Keywords:** modern technologies, social media, social networking platform, physical activity, wearable technologies

## Abstract

Several factors that follow the development of society affect physical inactivity, which primarily includes the development of technology and digitalization and the increasing choice of unhealthy lifestyle habits. However, certain shifts in the fitness industry have been noted in the last decade. The development of wearable technologies and artificial intelligence is one of the leading fitness trends and undoubtedly represents the future of the fitness industry. On the other hand, the significant influence of social media and networks affects the development and attitudes of people related to physical activity. Therefore, this review paper evaluates the advantages and disadvantages of wearable technologies and artificial intelligence, the positive and negative effects of social networks, and points out the problems accompanying these new fitness trends. The development of fitness trends follows humanity's needs, and one of the biggest challenges is incorporating these novelties in a mission to improve physical activity levels worldwide.

## Introduction

Physical activity (PA) is a generator for the improvement of quality of life. PA provides a broad spectrum of health benefits, including the decreased risk of early death, coronary heart disease, stroke, hypertension, type 2 diabetes, cancers, weight gain, risk of dangerous falls, anxiety, and cognitive decline (1, 2). Moreover, relatively robust scientific evidence indicates that exercise supports functional capacity in older adults, enhances sleep quality, and decreases the hazard of hip rupture and osteoporosis (3, 4). World Health Organization (WHO) reported that the minimum quantity and quality of PA that needs to be archived is 150 min/week of moderate PA or 75 min/week of vigorous-intensity (5). This only represents the minimum equality of time that one needs to be active, but promoting an even more physically active lifestyle could fill the gap and contribute even more to subjects' health and wellbeing (6).

However, people do not engage in this minimum amount of PA recommended. At this moment, we are facing the pandemic of physical inactivity (PI) and obesity (7). Around 31.1% of adults are physically inactive worldwide, with ratios ranging from 17% in Southeast Asia to about 43% in the Americas and the eastern Mediterranean (8). It was also noted that inactivity raises with age and is more represented among the female population and high-income countries (9). PI was recognized as the fourth leading risk factor for non-communicable diseases and responsible for more than 3 million preventable deaths (10). Additionally, PI also causes economic costs. For example, ~$50 billion are spent on healthcare systems worldwide yearly, while death attributable to PI costs another $13.7 billion in productivity losses (11).

Several factors influence these results of creating inactive, sedentary community, including the development of technology, digitalization, decrees in motivation, decrease of active commuting, higher rate of depression and anxiety, to name a few. In addition, the consequence of growing levels of PI might be caused by system-level factors (e.g., built environment) influencing PA behavior. It must be recognized that our obesogenic and PI-promoting environments partly cause inactivity. One of the leading challenges of society is how to motivate people and create adherence and discipline to exercise more and change their lifestyle behaviors. Different factors can influence adherence and motivation to exercise. Interestingly increasing influence of Social networks (media) can perhaps represent the silver bullet in closing the gap between PI and PA. Recently, Lakićević et al. (12) published an interesting opinion paper on the use of novelties as a crossroads in fitness. Although the authors of this study approached the problem from several perspectives; the role of the media, social networks, influencers, and novel fitness trends that follow the technological development of society and modern trends remains unclear. Therefore, this mini-review aims to critically evaluate the current trends in fitness their mutual impact on society, and give considerations and future directions of fitness.

## Shifting in Fitness Trends

Since 2006, the American College of Sports Medicine (ACSM) has administered an annual worldwide survey of fitness trends (13). This is provided in a mission to help fitness professionals make conceptual decisions to support customer engagement through a positive exercise experience. Although the fitness industry is continuously growing worldwide, PI and obesity epidemics have been at the highest rate in human history (14, 15). In addition, along with the promotion of PA as a key factor in all age populations, the role of fitness experts in fitness has been rapidly and continuously changing (16). This need for novelty has been followed by shifting in fitness for the last 15 years. For example, at the beginning of the twenty-first century, PA was recognized as the ultimate medicine, and there was growing interest in physical exercise (17). Therefore, at the top of fitness trends from 2006 to 2010 we found: promotions of educated and experienced fitness professionals, training for children and obesity, personal training, strength training, among others. Later, high-intensity interval training (HIIT) attracted extensive attention, which is still one of the leading fitness trends. Finally, as mentioned previously, wearable technologies are currently the leading fitness trend and represent the future of fitness industry development.

Excluding wearable technologies, performing new and challenging workouts could increase your enjoyment and interest while enhancing new abilities. Moreover, the need for novelty may represent a need for something that stands out of the routine (18). Exercise adherence could be increased by improving intrinsic motivation, finally resulting in better health outcomes (19). Happiness's underlying aspects fall into two dimensions: endogenic factors and exogenic factors. For the exerciser, PA must provide health benefits and emotional satisfaction. In theory, wellness can be defined as finding a balance between dimensions of life: emotional, spiritual, intellectual, physical, social, and environmental (20). Additionally, emotional wellness can be described as a “person's ability to cope with daily circumstances and to deal with personal feelings in a positive, optimistic, and constructive manner” (21). In order to greater adherence to the exercise model, the emotional aspect must not be neglected (22) New fitness trends must incorporate physiological and psychological aspects in order to attract the attention of exercisers. And it is of key importance that fitness trends follow also technological trends.

## Modern Technologies in Fitness

Following modern trends, there are some indications that fitness and physical exercise are changing. There is a certain shift in fitness trends. Based on ACSM leading fitness trend list (13), wearable technology has been the no. 1 trend since it was first introduced on the survey in 2016, except for 2018 (no. 3) and 2021 (no. 2). In the category of wearable technologies, it can be induced all activity trackers, smartwatches, GPS devices most usually used for counting steps, tracking heart rate, calories spent, activity levels, sleeping quality, and many more. It is estimated that the market for this industry is about $100 billion, with only growing potential (13).

The use of wearable technologies has been widely tested in the last 10 years, while the results of these studies have demonstrated mixed effectiveness. Few studies evaluated the effectiveness of wearable devices' on weight loss outcomes and the practice of PA. In the study by Fazzino et al., (23) a significant improvement in moderate-to-vigorous physical PA was noted after 6 months of intervention. In addition, participants maintained higher PA levels after 18 months of follow-up. Similar findings were found in the study by Chiang et al. (24) who evaluated the usage of wearable devices and adherence to PA, more precisely, step counting. After 2 years, the intervention group had statistically significantly percentage of total body weight loss compared to the control group. In addition, Findings in the population of young adults with a BMI between 25 and <40 showed that the addition of a wearable technology device to a standard behavioral intervention resulted in less weight loss over 24 months (25). Moreover, one study promised improved PA levels among medical students using the Fitbit vs. control (26). Contrary to these findings, the combination of smartwatches and health education courses did not enhance PA levels or reduce sedentary behavior among college students (27). In addition, 12 weeks of intervention with wearable technologies did not change the nutritional status or level of PA compared to the control group (28). These diverse observations mirror wearable technology randomized trials in overweight adults, the elderly population, and post-menopausal women (29–31).

One of the concerns of wearable technologies and smartwatches is their accuracy in measuring PA. This leaves room for improvement, but on the other hand, the current accuracy of the device is at a high level considering the general population's needs. The precision in step counting and self-observed steps using wearable technologies differed slightly, while smartwatches were even more accurate [for more, see review (32)]. The accuracy of wearable technology devices can range between 79.8 and 99.1%, while the coefficient of variation (precision) ranges between 4 and 17.5% (33). Wearables' different features and technology could explain variations in the accuracy of wearable devices. The precision of activity trackers and smartwatches will grow, and innovations in the field of technology may provide us with new possibilities. The development of wearable technologies is undoubtedly one of the growing trends in fitness and can represent a strategy to increase PA among the general population.

Artificial intelligence (AI) and associated computational techniques have recently become the new frontier in developing the landscape of fitness, health care, and promotion of PA (34). For example, AI chatbots, also called virtual conversational agents, engage in conversation techniques to facilitate natural language dialogues with users employing speech, text, or both to simulate human in-person communication (35, 36). Several studies evaluated the effectiveness of AI chatbots on healthy behaviors. For example, an AI chatbot called “Assistant to Lift your Level of activitY” (Ally) increased step-goal achievement (37). However, 30% of participants dropout throughout the study, suggesting a challenge for the chatbot's capability to engage participants. Several other chatbots have shown potential to improve dietary habits (38), promote self-reflection (39), and weight management (40). However, several issues are noted in the mentioned studies. For example, there are unanswered questions about ethical concerns regarding transparency, privacy, and potential algorithmic biases. In addition, details of the development of the chatbot program were not discuses in the studies. AI indeed represents the future directions in modern technologies usage in fitness, and it will be even more abundant in coming years.

## The Role of Social Media and Influences on Physical Fitness

Social media and social networking platform usage have exponentially grown in the last 10 years. Social media can be defined as “websites and computer programs that allow people to communicate and share information on the internet using a computer or mobile phone“ (41). In addition, these mediums can help promote healthier behaviors by providing users with the opportunity of learning (42). Through this type of media, YouTubers and influencers have the most influence.

A youtuber can be defined as someone who makes and appears in videos on the website, while an influencer can be defined as someone who affects or changes other people's behavior (43). Fitness is one of the topics covered by YouTube and Instagram users. Through their channels, fitness influencers describe their daily routines, give advice on exercise, diet, propose online coaching and free workouts, and generally (tik) talk about healthy lifestyles and living habits. Some youtube influencers have millions of audiences and represent a powerful and influential medium in transmitting the information. The growing influence of Internet has its foundations in psychology as well.

Earlier media models involved passive consumers theory, where consumers simply absorb media content (44). However, later theories suggest that the media is interactive where the audience actively chooses the information to process based on their own ideas and beliefs (45). This way, the media would strengthen the ideas and behavior instead of modifying them. Internet allows viewers to choose the content according to their preferences and beliefs. Also, entertainment is an integral aspect of the individual level, so discovering innovative practices to engage in PA that children/adolescents enjoy is essential and could incorporate online PA class, performing viral dances and challenges (e.g., TikTok), or PA gaming (46). Due to these possibilities, Internet has become the leading medium in the transmission of information, especially among the younger population.

Prior research reports that higher exposure and greater attention to health in the media could lead to broader knowledge concerning a healthy lifestyle and healthier behavior (47, 48). Contrary to professional athletes, the audience senses influencers as peers and could relate to them (49), and it is this model and this determinant that has proven to be the critical factor in conveying messages (50). Through their channels, fitness influencers promote healthy lifestyle habits and PA. For example, on such channels l, coaching videos, exercise tutorials, motivational speeches, videos featuring past and current experiences of the influencer, can be regularly found (51). Following the viewer's theory of active involvement in media selections based on viewer attitudes and beliefs, it is rational to presume that viewers and followers of fitness influencers are usually interested in healthier lifestyles and fitness, so this type of content can positively impact their overall health behaviors. However, there are also negative sides to social media. For example, some studies have found the unfavorable effects of social networks on body image, body satisfaction, and eating disorders (52, 53). Moreover, it is questionable whether online sharing and promotion of PA throughout website content influences the actual PA performed (54, 55). Understanding how to use social networks and media to promote PA behaviors is limited (56). Moreover, there are limited data that evaluate the role of the influencers in promoting healthier behaviors (57).

Content designed to promote PA, healthy lifestyle habits, and a positive attitude toward fitness are likely to impact the viewer/consumer of online content positively. However, increased attention and careful consideration are advisable while using social networks and youtube channels to inspire the transition toward healthy behaviors. It is a mistake to assume that watching fitness channels can increase PA. Moreover, it could even reduce the intentions of the viewers to exercise (57).

## Conclusion

PI was defined as a global pandemic in the 2012 Lancet series on PA and health. PI has hazardous health and economic consequences. One of the most important challenges to modern societies is increasing awareness of PA's importance and a healthy lifestyle. However, there is no silver bullet for PA promotion. Although many efforts have been made to promote PA, the global level of PI is still high. Therefore, it is essential to establish some goals that will follow the needs and development of modern society. On the one hand, the growing influence of influencers and social networks on the promotion of PA is a good strategy, while on the other hand, a cause for concern. The question is how to limit the influence of influencers due to the possible harmful effects of the content of their channel and the accuracy of the information itself. On the other hand, leading healthcare organizations should take the initiative and start educating fitness influencers who impact a considerable number of people, thus providing Internet users with more accurate information. In the end, the development of AI does indeed represent the future of fitness, and we will be able to assess the real benefits and consequences of these technologies in the assessments that lie ahead.

With this short review additional questions arise and need to be answered:

(1) How can wearable technologies be popularized and implemented in PA interventions worldwide?
(2) How can social networks implement PA advertising/ promotion strategies at scale?
(3) How can the next wave of technological improvements such as AI be integrated into large-scale PA promotional programs.

Enhancing global health by increasing PA will demand both advancements in knowledge and a more significant commitment of resources.

## Author Contributions

VŠ, NT, and PD: conceptualization and writing–original draft preparation. MR and NT: methodology. IM, SP, MR, and NT: writing–review and editing. PD: visualization and supervision. VŠ and PD: funding acquisition. All authors have read and agreed to the published version of the manuscript.

## Funding

This work was supported by the Provincial Secretariat for Higher Education and Scientific Research (142-451-2597/2021-01/2).

## Conflict of Interest

The authors declare that the research was conducted in the absence of any commercial or financial relationships that could be construed as a potential conflict of interest.

## Publisher's Note

All claims expressed in this article are solely those of the authors and do not necessarily represent those of their affiliated organizations, or those of the publisher, the editors and the reviewers. Any product that may be evaluated in this article, or claim that may be made by its manufacturer, is not guaranteed or endorsed by the publisher.
